# Compact coherence enhancement by subharmonic self-seeding in X-ray free-electron laser facilities

**DOI:** 10.1107/S1600577518000395

**Published:** 2018-02-19

**Authors:** Eduard Prat, Sven Reiche

**Affiliations:** a Paul Scherrer Institut, CH-5232 Villigen PSI, Switzerland

**Keywords:** free-electron laser, self-seeding, X-ray coherence, simulations

## Abstract

X-ray free-electron lasers (FELs) are cutting-edge scientific tools able to generate transversely coherent radiation with very high power and ultra-short pulse durations. The self-seeding mechanism has been proven to increase the longitudinal coherence of the FEL radiation but its efficiency could be significantly improved, especially for soft X-rays. This paper proposes the enhancement of the performance of self-seeding by combining it with the harmonic generation mechanism. In particular, by starting the process with a subharmonic of the wavelength of interest, the coherence of the produced radiation is improved, the undulator beamline becomes more compact, and the monochromator realization is simplified. Numerical simulations for SwissFEL are presented showing that the method can be employed, within a given space, to increase the spectral brightness by one order of magnitude or more with respect to standard self-seeding. This coherence enhancement will be fundamental for many photon science applications and techniques such as resonant inelastic X-ray scattering.

## Abstract 2

A simple method to significantly enhance the coherence of X-ray free-electron laser (FEL) pulses by combining self-seeding and the harmonic generation mechanism is presented. This work is a fundamental step towards obtaining fully coherent FEL pulses, solving in this way a critical problem in the FEL field and its multidisciplinary scientific applications. In particular, the coherence improvement will be extremely beneficial for many photon science applications requiring a small bandwidth such as resonant inelastic X-ray scattering, therefore opening new areas of research and playing a crucial role in the future of all scientific users of the FEL facilities.

## 1. Introduction   

X-ray free-electron lasers (FELs) are modern research instruments capable of generating radiation with wavelengths down to the ångstrom level, peak powers of tens of gigawatts or larger, and pulse durations shorter than tens of femto-seconds (Emma *et al.*, 2010 ▸; Ishikawa *et al.*, 2012 ▸; Amann *et al.*, 2012 ▸; Allaria *et al.*, 2013 ▸; Ackermann *et al.*, 2007 ▸). Most of the state-of-the-art FEL facilities are based on the self-amplified spontaneous emission (SASE) process (Kondratenko & Saldin, 1980 ▸; Bonifacio *et al.*, 1984 ▸), which starts from the electrons’ shot noise. As a result of this, the radiation obtained from the SASE-FELs is not fully coherent over the longitudinal extent of the bunch, with typical relative bandwidths in the 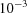 to 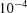 level.

**Figure 1:**
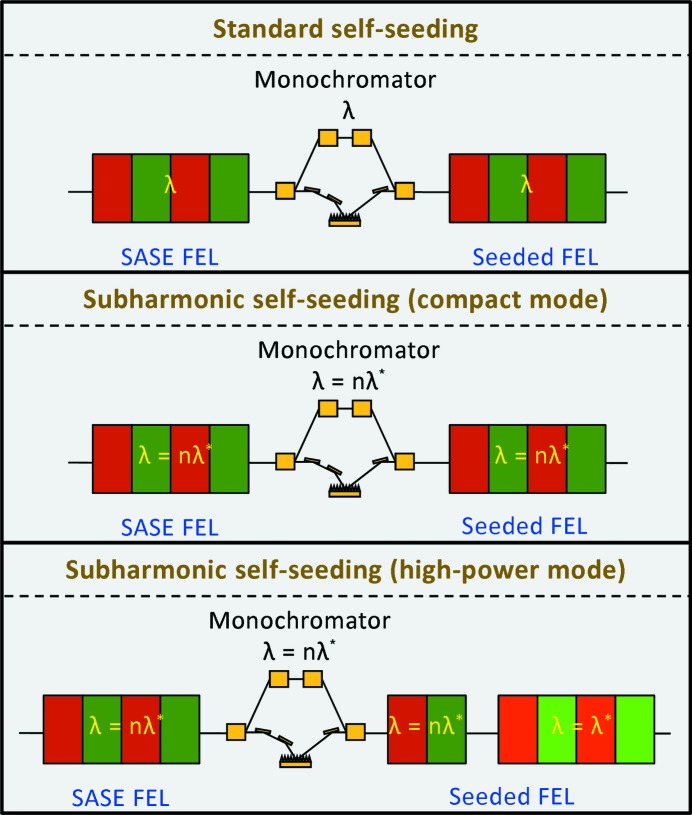
Schematic layouts of the standard self-seeding scheme (top) and the two implementations of the proposed method (center and bottom).

Many FEL users need to filter the SASE signal to achieve a narrower bandwidth and improved longitudinal coherence. Utilizing a monochromator, however, reduces the available number of photons. Instead, seeding techniques are a better choice to increase the coherence of the SASE-FEL radiation. The coherence enhancement also helps improve the efficiency of tapering the undulator field to maximize the extracted FEL power (Kroll *et al.*, 1981 ▸; Fawley *et al.*, 2002 ▸). Seeding with external lasers, directly with a high-harmonic-generation source (Togashi *et al.*, 2011 ▸; Ackermann *et al.*, 2013 ▸) or with more complex setups such as the high-gain harmonic generation (Yu, 1991 ▸; Allaria *et al.*, 2012 ▸, 2013 ▸) or the echo-enabled harmonic generation schemes (Stupakov, 2009 ▸; Zhao *et al.*, 2012 ▸; Hemsing *et al.*, 2016 ▸), is presently limited to around 0.3 keV (Allaria *et al.*, 2013 ▸) and going beyond is challenging due to wavelength limitations of the available lasers and noise degradation problems (Saldin *et al.*, 2002 ▸). For the X-ray regime, one can employ the self-seeding mechanism, where, as shown schematically in the top of Fig. 1 ▸, the SASE radiation, generated in a first undulator section, passes through a monochromator to be later amplified in a second undulator stage (Feldhaus *et al.*, 1997 ▸; Saldin *et al.*, 2001 ▸; Geloni & Saldin, 2010 ▸; Ratner *et al.*, 2015 ▸; Amann *et al.*, 2012 ▸). Self-seeding has been experimentally demonstrated for both soft and hard X-rays at the Linac Coherent Light Source (LCLS) at SLAC (Ratner *et al.*, 2015 ▸; Amann *et al.*, 2012 ▸). However, the efficiency of self-seeding should be improved further, especially in the soft X-ray regime where only a moderate resolving power between 2000 and 5000 has been demonstrated (Ratner *et al.*, 2015 ▸). This is not sufficient to extend the coherence on the entire bunch for wavelengths of a few nanometers or shorter; ideally the coherence time should be given by the electron bunch length.

The fundamental wavelength of the FEL radiation is given by (Bonifacio *et al.*, 1984 ▸)
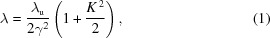
where 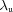 is the period length of the undulator, γ is the Lorentz factor of the electron beam, and *K* is the undulator field parameter. In a planar undulator, the FEL process also occurs at the odd harmonics of the fundamental wavelength (Vignola *et al.*, 1985 ▸; Bonifacio *et al.*, 1990 ▸; Freund *et al.*, 2000 ▸; Huang & Kim, 2000 ▸; Tremaine *et al.*, 2002 ▸; Saldin *et al.*, 2006 ▸; Ratner *et al.*, 2011 ▸; Murphy *et al.*, 1985 ▸; McNeil *et al.*, 2006 ▸; Schneidmiller & Yurkov, 2012 ▸).

Here we propose to improve the self-seeding scheme by combining it with the harmonic generation mechanism. Specifically, we put forward the idea of driving the self-seeding with the subharmonic of the wavelength of interest, thus our scheme may be called *subharmonic self-seeding*. As a result, the coherence of the radiation will be higher, the undulator length will be reduced, and the requirements on the self-seeding monochromator will be relaxed. A higher longitudinal coherence will be beneficial for many photon science applications and techniques that require FEL radiation with very small bandwidth. For instance, it will improve the resolution of resonant inelastic X-ray scattering, a cutting-edge spectroscopic technique with a unique potential to probe fundamental excitations in complex materials (Ament *et al.*, 2011 ▸; Dell’Angela *et al.*, 2016 ▸). The method can be applied to both hard and soft X-rays; here we will focus on the soft X-ray case.

## 2. Description of the scheme   

Fig. 1 ▸ provides schematic layouts of the standard self-seeding scheme and the two possible implementations of our setup, one to maximize the radiation power (high-power mode) and another to minimize the required undulator length (compact mode). For both modes, as in the standard self-seeding, there is a first stage that produces SASE-FEL radiation, a monochromator that improves the coherence of the SASE-FEL light, and a second stage that amplifies the monochromatic radiation to a higher power level. In our case, however, the undulators of the first stage and the monochromator are tuned to a wavelength λ which is a subharmonic of the wavelength of interest 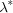, *i.e.* 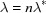, where *n* is the harmonic number. Before the monochromator, SASE-FEL radiation is generated for the subharmonic (λ) and its harmonics or the wavelength of interest (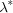). In the compact mode, the monochromator is followed by a series of undulators tuned to the subharmonic (λ), which produce seeded FEL radiation for both the subharmonic (λ) and the wavelength of interest (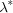) in the nonlinear regime up to saturation. This causes a fast power growth of the radiation at the wavelength of interest, which is, however, limited to a lower final value than in the high-power mode. In that case, the monochromator is followed by an initial row of undulators tuned to the subharmonic (λ), which generate seeded FEL radiation for both the subharmonic (λ) and the wavelength of interest (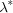) well before the FEL process reaches saturation. Afterwards, the beam is sent to undulator modules tuned to the wavelength of interest (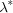), which boost the radiation at that wavelength to a higher power level than in the compact implementation. In the high-power implementation, the requirement of having undulators tuned to the subharmonic and the wavelength of interest can be achieved either with variable-gap undulators able to provide different undulator fields, *K*, or with two undulator types with different periods, 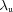.

The key idea of our scheme is that the coherence time given by the monochromator is much longer with the subharmonic radiation, assuming comparable resolving powers for both wavelengths. In a second step, the coherent radiation at the subharmonic (λ) will drive in the second stage coherent radiation at its harmonics or the wavelength of interest (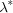). In the compact mode, the coherence time of the subharmonic will be imprinted to the wavelength of interest *via* the nonlinear harmonic generation process and, consequently, the relative bandwidth will be improved by a factor equal to the harmonic number (Pellegrini *et al.*, 2016 ▸). In the high-power mode, the harmonic process in the undulators after the monochromator enters the nonlinear regime but does not reach saturation. This is to avoid a spectrum deterioration within the undulators tuned to the wavelength of interest due to the onset of sideband instabilities (Kroll & Rosenbluth, 1980 ▸) which are enhanced in the harmonic conversion process. Consequently, for the high-power implementation the increase in coherence will be reduced in comparison with the compact mode. The shift from the undulators tuned to the subharmonic to those at the wavelength of interest needs to be done at the optimum location for best performance.

In addition to the coherence improvement, both implementations of the proposed scheme will be more compact than the standard self-seeding: the first stage will be significantly reduced because it is tuned to the subharmonic with a much shorter gain length than if tuned to the wavelength of interest. In our scheme, the monochromator is employed to produce FEL radiation outside its operational range (a monochromator working at 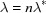 can provide radiation at 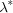). Therefore, another advantage of subharmonic self-seeding is that the realization of a monochromator to cover a certain wavelength range becomes easier. As an example, a monochromator operating between 1.5 and 4.5 nm is sufficient to produce FEL radiation down to 0.5 nm when using the third subharmonic. This particular example illustrates that a monochromator suited for soft X-rays can be used in our scheme to generate hard X-ray FEL pulses. Our method is intended to be useful for shorter wavelengths in the nominal tuning range of the FEL. Taking the previous example, to cover a wavelength range between 0.5 and 4.5 nm, standard self-seeding would be employed between 1.5 and 4.5 nm, while subharmonic self-seeding would be used between 0.5 and 1.5 nm.

The main advantage of the high-power mode, in comparison with the compact implementation, is that the resulting radiation may have higher power and spectral brightness. Moreover, filtering the radiation of the subharmonic is much easier, since the power level of the subharmonic is much lower and its radiation has a different source point than the one produced at the wavelength of interest. The compact mode, however, does have its own benefits: it requires a shorter undulator beamline, the final coherence is higher, it can be realized with a single type of fixed-gap undulators, and it allows expanding the wavelength range of the FEL facility (producing radiation at a certain wavelength only requires generating FEL radiation at the subharmonic of that wavelength).

Our method resembles three earlier proposals. First, Geloni and co-workers proposed for the first time the high-power mode of subharmonic self-seeding for hard X-rays using bunching at the second harmonic (Geloni *et al.*, 2011 ▸, 2015 ▸). In our case we propose using the radiation naturally generated at the odd harmonics and we focus our analysis on the coherence enhancement by starting the self-seeding mechanism with the subharmonic radiation. Second, the scheme described by Schneidmiller & Yurkov (2012 ▸, 2013 ▸) and Schneidmiller *et al.* (2017 ▸) is similar to the second stage of the high-power implementation of our scheme, but implemented for SASE-FEL pulses to achieve a moderate improvement of their coherence and saturation length. While in that case the nonlinear coupling is avoided in order not to ruin the spectral bandwidth, in our proposal it is explicitly utilized to transfer the coherence properties to the wavelength of interest. Third, Emma *et al.* (2017*a* ▸) suggested combining the self-seeding with harmonic generation, but with the monochromator directly tuned to the wavelength of interest. In our case, since the monochromator is tuned to the subharmonic, the final coherence of the resulting radiation is significantly better.

## 3. Simulations   

We will now discuss the performance of the subharmonic self-seeding scheme by means of an example for the future soft X-ray beamline of SwissFEL (Milne *et al.*, 2017 ▸; Prat *et al.*, 2016 ▸), expected to generate FEL radiation between 0.65 and 5 nm. We have performed FEL simulations with the code *Genesis 1.3* (Reiche, 1999 ▸) with the following electron-beam parameters: the energy is 3.15 GeV, the current profile is flat with a peak value of 3 kA and a total bunch length of 20 µm, the normalized transverse emittance is 300 nm, the slice energy spread is 350 keV [root mean square (RMS)], and the average β function is about 5 m. The undulator beamline will initially consist of 16 undulator modules, with possible extensions in future upgrades. Each undulator module has an undulator period of 38 mm and a total length of 2 m, and the undulator field parameter *K* can be scanned between 0.8 and 3.5. The space between undulator modules is about 0.7 m. We assume that the energy spread is not degraded in the first stage before the monochromator, which could be achieved by using a fresh bunch in the second undulator stage (Emma *et al.*, 2016 ▸, 2017*a* ▸,*b* ▸; Lutman *et al.*, 2016 ▸; Reiche & Prat, 2016 ▸; Prat *et al.*, 2015 ▸). Our system, however, is rather robust against larger energy spreads: we have simulated that the FEL power in the harmonics only drops by about 25% when increasing the energy spread from 350 to 1000 keV. Table 1 ▸ shows the parameters used in the simulations presented here.

**Figure 2:**
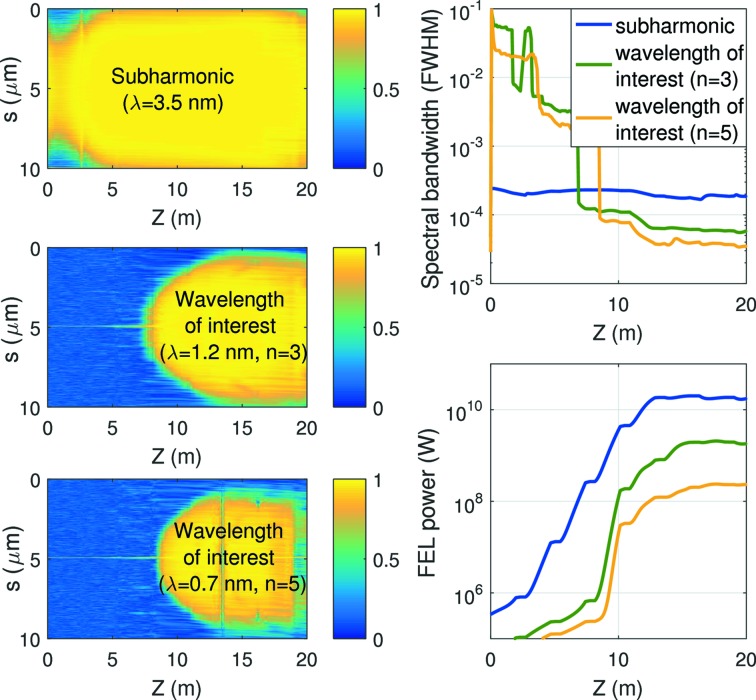
Left: first-order correlation function 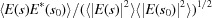 (where *E* is the radiation field) with respect to the bunch center 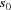 as a function of the longitudinal position *s* along the bunch and the bunch position *Z* along the undulator beamline for the radiation at the subharmonic (top) and at the wavelengths of interest (center and bottom). Right: relative bandwidth (top) and FEL power (bottom) along *Z* for the different cases.

First, we study the transfer of coherence to the harmonics. We use as an input coherent radiation at a wavelength of 3.5 nm. Fig. 2 ▸ shows the evolution of the first-order correlation function, the spectral bandwidth, and the FEL power for the subharmonic (fundamental) and the wavelengths of interest (third and fifth harmonics). The results are calculated for 50 different shots. The input seed has a Gaussian shape with a peak power of 1 MW and a length of 3 µm (RMS). It can be seen that the coherence of the radiation at the wavelengths of interest is quite poor at the beginning of the beamline, but improves after a certain distance driven by the coherent radiation at the subharmonic. The final relative bandwidth of the radiation at the wavelength of interest is, as expected, better than the one at the subharmonic by a factor approximately equal to the harmonic number. This mimics the second stage of the compact mode. In the case of the standard self-seeding configuration, working directly at the wavelength of interest, the length of the seed pulse would be shorter by a factor equal to the harmonic number.

**Figure 3:**
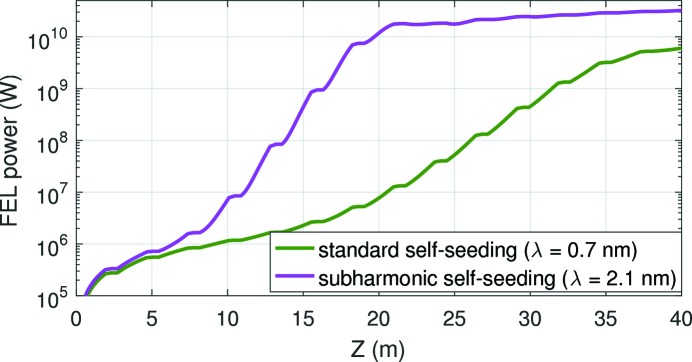
SASE-FEL power along the undulator beamline for the standard and the subharmonic self-seeding schemes.

We have performed simulations for the full setup with a wavelength of interest of 0.7 nm. We have considered all methods shown in Fig. 1 ▸, with 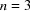 for the subharmonic self-seeding. To illustrate the performance of the scheme in the first stage before the monochromator, Fig. 3 ▸ shows the SASE-FEL power along the undulator beamline for the standard self-seeding configuration, for which the undulators are tuned to the wavelength of interest (0.7 nm corresponding to 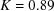), and for the subharmonic self-seeding, for which the undulators are tuned to the third subharmonic (2.1 nm corresponding to 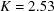). The results are averaged over five runs with different seeds for the generation of the electrons’ shot noise. No tapering of the undulator field (Kroll *et al.*, 1981 ▸) is applied. The statistical errors, not shown in the plot, amount to less than 10%. For the subharmonic self-seeding, the FEL process reaches saturation in about eight modules at a power around 20 GW, while for the standard self-seeding we would need almost twice the length to saturate at a power of 6 GW. This illustrates the first advantage of our method: it is possible to send more radiation power to the monochromator with much fewer undulator modules.

We have simulated the second stage after the monochromator taking as an input seed coherent radiation with a power of 1 MW. We adopt a second stage consisting of 16 undulator modules and assume that the resolving power of the monochromator is 5000 for all radiation wavelengths. This is roughly consistent with state-of-the-art monochromators but it is conservative in the sense that the resolving power of soft X-ray monochromators is typically larger for longer wavelengths (see, for instance, Cocco *et al.*, 2013 ▸). As an example, for the experiment presented by Ratner *et al.* (2015 ▸), the resolving power of the grating monochromator was 4800 for a photon energy of 1 keV (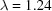
 nm) and 6800 for 0.5 keV (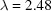
 nm) (Cocco *et al.*, 2013 ▸). Therefore, the coherence improvement using subharmonic self-seeding could be better in reality than that shown here. The coherence length for a radiation wavelength of 0.7 nm and a resolving power of 5000 is 3.5 µm. Since this is much shorter than the full bunch length (20 µm), one could potentially achieve smaller bandwidth by increasing the coherence. We assume the monochromator to have an efficiency in terms of radiation power of 0.1%, which is consistent with previous work (Ratner *et al.*, 2015 ▸). Thus, starting with 1 MW of coherent signal would require 1 GW of SASE-FEL radiation from the first stage. For the subharmonic self-seeding, this would be achieved in six undulator modules, while 15 m more of undulator beamline would be required for standard self-seeding.

**Figure 4:**
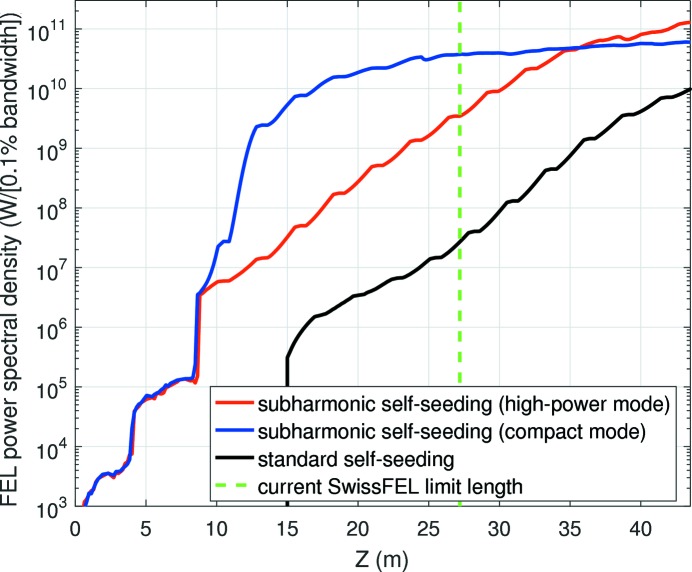
FEL power spectral density along the undulator beamline for the standard self-seeding and the two modes of the subharmonic self-seeding.

Table 2 ▸ and Fig. 4 ▸ show the simulation results of the second stage after the monochromator. For the standard self-seeding configuration, we shift the beginning of the second stage to take into account that 15 m more would be required to reach 1 GW of FEL power in the first stage. Table 2 ▸ shows the final FEL average power, the relative bandwidth at saturation and the final power spectral density at the wavelength of interest for the three cases considered. The power spectral density, equivalent to brightness, is calculated as the FEL average power over the spectral bandwidth (FWHM values) in units of W (0.1% bandwidth)^−1^. For the subharmonic self-seeding configurations, we also indicate the FEL power at the subharmonic radiation. Fig. 4 ▸ displays the FEL power spectral density along the beamline. The results are averaged over 25 seeds. The statistical errors, not indicated in the figure and the table, are well below the 10% level. For each case we have optimized the value of the undulator field before saturation to minimize the spectral bandwidth and the linear taper amplitude after saturation to maximize the FEL power. The optimum taper is about 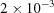 for the standard self-seeding, 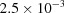 for the high-power mode of the subharmonic self-seeding, and 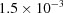 for its compact implementation. For the high-power mode, we have also optimized the number of modules tuned to the subharmonic radiation: in our case, the best performance is obtained when three modules are tuned to the subharmonic (2.1 nm) and the rest are in resonance with the wavelength of interest (0.7 nm). Having rather short undulator modules, as in our case, facilitates the optimization of the location where the undulator resonance condition is changed from the subharmonic to the wavelength of interest.

The table and the figure illustrate that higher powers and smaller bandwidths can be obtained with subharmonic self-seeding. In comparison with the standard self-seeding configuration, the power spectral density or brightness at the end of the beamline is 13 (6) times higher for the high-power (compact) implementation. The high-power mode reaches power spectral densities at the end of the beamline around a factor of two higher than the compact implementation, but the latter is able to achieve an already decent performance in a much shorter distance. For instance, for the initially available space in SwissFEL (16 modules in total, 10 in the second stage), the compact mode gives a power spectral density of about 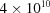
 W (0.1% bandwidth)^−1^, about ten times higher than in the high-power implementation (and in any case much higher than for the standard self-seeding). The coherence improvement with respect to the standard self-seeding is about a factor of three (two) for the compact (high-power) mode of the subharmonic self-seeding. The bandwidth reduction in the high-power mode is not equal to the harmonic number because, as explained before, the shift from the undulators tuned to the subharmonic to those tuned to the wavelength of interest needs to be established before the bandwidth reduction is fully achieved, or else sideband instabilities spoil the spectrum. Finally, we observe in Table 2 ▸ that the FEL power of the subharmonic radiation is almost negligible for the high-power mode (0.05 GW), while it is considerable for the compact implementation (82.7 GW). This, as explained before, will render filtering the subharmonic radiation much easier in the high-power mode.

We have also calculated the case for the standard self-seeding configuration where the number of undulator modules in the second stage equals that used for the subharmonic self-seeding (16). After taper optimization the final FEL power would be equivalent to the one obtained with the higher-power mode of our scheme (around 17 GW), but the coherence and brightness would still be a factor of two worse than with subharmonic self-seeding. Moreover, the facility would need 15 m more of undulator beamline, equivalent to five or six undulator modules.

## 4. Further possibilities   

The performance of the scheme could be further improved by working at the fifth or higher harmonics. For higher harmonic numbers, however, the high-power mode becomes more sensitive to sideband instabilities and the power is reduced in the compact implementation. Moreover, one could use progressively several fresh fractions of the bunch to increase the power of the produced radiation, as proposed by Prat *et al.* (2015 ▸) with a spatially chirped beam. In addition, our method could also be implemented to improve the efficiency of the high-brightness SASE scheme, a compact alternative to self-seeding to increase the coherence of the FEL radiation by delaying the electrons with respect to the photons (Thompson *et al.*, 2010 ▸; McNeil *et al.*, 2013 ▸; Wu *et al.*, 2013 ▸). The detailed analysis of these aspects is, however, beyond the scope of this work.

## 5. Conclusion   

In conclusion, a method is presented that improves the performance of the self-seeding in FEL facilities by combining it with the harmonic generation mechanism: brighter radiation can be obtained within a shorter undulator beamline, and the efficiency of the monochromator is effectively enhanced. It has been shown with numerical simulations that, for a given undulator beamline, the increase in spectral brightness is of one order of magnitude or more with respect to the standard self-seeding scheme. The compact implementation of the subharmonic self-seeding could be readily tested at LCLS; for the high-power mode an FEL facility with an X-ray monochromator and variable gap undulators, such as the future soft X-ray beamline of SwissFEL, is required.

## Figures and Tables

**Table 1:** Simulation parameters

Parameter	Value
Electron energy	3.15 GeV
Electron current profile	Flat
Electron peak current	3 kA
Electron bunch length	20 µm
Normalized transverse emittance	300 nm
RMS uncorrelated energy spread	350 keV
β-function	5 m
Undulator module length	2 m
Undulator period	38 mm
Undulator field parameter *K*	0.8–3.5

**Table 2:** Simulation results

	Subharmonic self-seeding		
Parameter	High-power mode	Compact mode	Standard self-seeding
FEL power: wavelength of interest (λ = 0.7 nm)	16.6 GW	3.0 GW	1.7 GW
FEL power: subharmonic (λ = 2.1 nm)	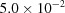 GW	82.7 GW	0 GW
Relative bandwidth (λ = 0.7 nm)	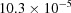	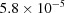	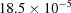
FEL power spectral density	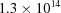 W (0.1% bandwidth)^−1^	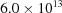 W (0.1% bandwidth)^−1^	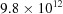 W (0.1% bandwidth)^−1^
